# Immunogenetic and Transcriptomic Evidence Implicating the NKG2D-MICA/MICB Axis in CALR-Mutated Myeloproliferative Neoplasms

**DOI:** 10.3390/cancers18132052

**Published:** 2026-06-24

**Authors:** Velizar Shivarov, Gergana Tsvetkova, Ilina Micheva, Evgueniy Hadjiev, Jasmina Petkova, Galia Madjarova, Milena Ivanova

**Affiliations:** 1 Department of Experimental Research, Medical University Pleven, 5800 Pleven, Bulgaria; 2 Department of Clinical Hematology, St. Sophia General Hospital, 1618 Sofia, Bulgaria; 3 Department of Clinical Hematology, Alexandrovska University Hospital, Medical University Sofia, 1431 Sofia, Bulgaria; 4 Department of Clinical Hematology, Saint Marina University Hospital, Medical University Varna, 9010 Varna, Bulgaria; 5 Department of Physical Chemistry, Faculty of Chemistry and Pharmacy, Sofia University “St. Kl. Ohridski”, 1164 Sofia, Bulgaria; 6 Department of Clinical Immunology, Alexandrovska University Hospital, Medical University Sofia, 1431 Sofia, Bulgaria

**Keywords:** myeloproliferative neoplasms, calreticulin (CALR), NKG2D, MICA, MICB, KLRK1, immunogenetics, immune surveillance

## Abstract

*Background/Objectives:* Immune surveillance is increasingly recognized as a modifier of myeloproliferative neoplasm (MPN) initiation and evolution, yet the contribution of the NKG2D receptor and its ligands MICA/MICB to CALR-mutated disease remains unclear. *Methods:* We performed high-resolution next-generation sequencing genotyping of MICA and MICB in 43 patients with CALR-mutated MPN (WHO 2022 criteria) and compared the allele and haplotype distributions with those of 156 healthy Bulgarian controls and 85 patients with JAK2 V617F-positive MPN. Associations were tested using age- and sex-adjusted additive generalized linear models; bi-locus haplotypes were evaluated using haplotype score methods. In a genotyped subgroup (35 CALR-mutated MPN patients and 105 controls), functional KLRK1 (NKG2D) polymorphisms were analyzed for haplotype-level associations. We also performed 700 ns molecular dynamics simulations of selected MICA variants in complex with NKG2D and reanalyzed publicly available single-cell RNA-sequencing data (GSE117826) and RNA-sequencing data from CRISPR/Cas9-edited CALR-mutant iPSC-derived megakaryocytes to evaluate MICA/MICB expression. *Results:* MICA*004:001 was significantly associated with CALR-mutated MPN versus controls (*p* = 0.004; Bonferroni-adjusted *p* = 0.047), while MICB*008:001 showed only nominal association. Exploratory haplotype analyses identified a MICA*009:01-MICB*004:001 haplotype associated with CALR-mutated status (*p* = 0.008) and a KLRK1 G-A-G-T haplotype (rs1049174-rs2617160-rs2246809-rs2617170) associated with increased CALR-mutated MPN risk (OR = 3.61; *p* = 0.029). Transcriptomic reanalysis indicated a higher fraction of CALR-mutant stem and progenitor cells expressing detectable MICA/MICB transcripts, and heterozygous CALR-mutant megakaryocytes exhibited higher MICA expression than the wild type. *Conclusions:* Together, these data support an exploratory immunogenetic and transcriptomic link between the NKG2D-MICA/MICB axis and CALR-mutated MPN, but direct protein-level and functional studies are required before mechanistic or therapeutic conclusions can be drawn.

## Simple Summary

Myeloproliferative neoplasms are blood cancers in which blood-forming cells grow abnormally. In some patients, these diseases are driven by mutations in the *CALR* gene. The immune system may help control early abnormal blood-cell clones, but the mechanisms involved are still not fully understood. In this study, we investigated whether genetic variation in the NKG2D–MICA/MICB immune-recognition pathway is linked to CALR-mutated myeloproliferative neoplasms. We found that one MICA allele, MICA*004:001, was more frequent in patients with CALR-mutated disease than in healthy controls. Additional exploratory analyses suggested possible associations involving MICB and KLRK1/NKG2D haplotypes. We also found transcriptomic evidence that CALR-mutant stem and progenitor cells may show altered MICA/MICB expression. Together, these findings suggest that the NKG2D–MICA/MICB axis may contribute to immune surveillance in CALR-mutated myeloproliferative neoplasms. However, larger studies and direct functional experiments are needed before firm biological or therapeutic conclusions can be made.

## 1. Introduction

Philadelphia chromosome-negative myeloproliferative neoplasms (MPNs) are paradigmatic multistep clonal disorders arising from hematopoietic stem cells (HSCs) and shaped by both cell-intrinsic lesions and the microenvironment [1]. The three major entities—essential thrombocythemia (ET), polycythemia vera (PV), and primary myelofibrosis (PMF)—share recurrent driver mutations in JAK2, CALR, and MPL, yet present with distinct morphology and clinical phenotypes [1]. Beyond oncogenic signaling, MPNs are characterized by a chronic inflammatory milieu that sustains myeloproliferation, contributes to symptom burden, and has therapeutic implications [2]. Growing evidence suggests that immune surveillance operates early in MPN pathogenesis and may constrain the expansion of nascent mutant clones [1]. Accordingly, immunogenetic variation in HLA genes [3,4,5,6], MICA/MICB [7], cytokine loci [8,9], and immune checkpoint genes [10] has been investigated as a determinant of disease susceptibility and progression.

HLA class I (HLA-I) alleles modulate immune control of MPN by determining which neoepitopes can be presented to cytotoxic T cells. Several HLA-I alleles are under-represented among patients with JAK2 V617F-mutated MPNs, consistent with a protective effect via T-cell immunosurveillance [4]. For example, HLA-B*35:01 (as well as HLA-A*02:01 and HLA-C*15:02) has been reported at a lower frequency in JAK2-mutated disease and can bind to a JAK2 V617F-derived peptide, supporting a mechanistic link between antigen presentation and clone elimination [4]. Conversely, MPN stem/progenitor cells may downregulate HLA-I expression, impairing neoantigen display and facilitating immune escape; interferon-alpha and JAK1/2 inhibition have been reported to restore HLA-I expression in this compartment, potentially increasing tumor visibility to the immune system [4].

Recent work further suggests that the host HLA repertoire can influence which driver mutation successfully establishes disease. In a recent immunogenetic analysis, HLA-C*06:02 was under-represented among CALR-mutated MPNs, consistent with efficient presentation of mutant CALR-derived peptides in carriers [6]. More broadly, the predicted capacity of an individual’s HLA-I alleles to present JAK2 V617F-derived versus CALR-mutant peptides showed an inverse relationship, supporting an HLA-mediated immunoediting model that may contribute to the mutual exclusivity of these driver mutations [6]. CALR-mutant hematopoietic cells also displayed lower expression of antigen-processing genes (e.g., *TAP1* and *CIITA*), consistent with downregulation of antigen presentation pathways [6].

The MHC class I-related molecules A and B (*MICA* and *MICB*) are stress-inducible ligands for the activating receptor NKG2D expressed on natural killer (NK) cells and subsets of T cells and are, therefore, poised to shape immunosurveillance in hematologic malignancies [11]. In JAK2 V617F-positive MPN, specific MICA alleles (e.g., MICA*008:01 and MICA*016) were reported at a lower frequency than in controls, and circulating soluble MICA was increased, consistent with ligand shedding as an immune-evasion mechanism [7]. While mature myeloid cells may upregulate MICB, MPN CD34+ stem/progenitor cells can downregulate MICA/MICB expression, potentially limiting NK cell recognition during disease establishment [7].

Here, we investigated whether genetic variation in *MICA/MICB* and the NKG2D receptor gene (*KLRK1*), as well as transcriptional regulation of *MICA/MICB* across CALR-mutant hematopoietic compartments, contributes to the pathogenesis of CALR-mutated MPNs.

## 2. Materials and Methods

### 2.1. Patients

We analyzed peripheral blood DNA from 43 patients with CALR-mutated, Philadelphia chromosome-negative MPN diagnosed according to the 2022 WHO criteria [12]. The MICA and MICB allele and haplotype distributions were compared with those of 85 patients with JAK2 V617F-positive MPNs from our prior cohort [4] and with those of 156 healthy Bulgarian controls from our immunogenetics of aging studies [13]. The demographic and clinical characteristics are summarized in Table 1. Because controls were younger than patients, age was included as a covariate in association models; sex was also included. All participants provided written informed consent. This study was approved by the Institutional Ethics Committee at the Medical University—Sofia under protocol 05/20 March 2025. All subjects provided informed consent in accordance with the Declaration of Helsinki.

### 2.2. DNA Extraction

Genomic DNA was extracted from sodium citrate peripheral blood (PB) using the IPrep (Life Technologies, Paisley, UK) automated system, as described previously [13]. Genomic DNA samples were stored at −20 °C until being genotyped.

### 2.3. Genotyping for MPN-Associated Mutations

Genotyping for JAK2 (including V617F), MPL, and CALR mutations was performed using direct sequencing and/or a bead-based liquid assay, as previously described [14,15,16].

### 2.4. MICA and MICB Genotyping and Association Analyses

*MICA* and *MICB* genotyping were performed on 43 CALR-mutant MPNs, 85 MPNs from JAK2 V617F-positive patients and 156 healthy controls by NGS whole-gene amplification using NGSgoR AmpX MICA with the MICB amplification kit (GenDX, Utrecht, The Netherlands). MICA and MICB genotypes were assigned by the NGSengine analysis software (GenDX, Utrecht, The Netherlands) and the IPD-IMGT/HLA database, as described previously [17]. Considering the difference in terms of age between patients and controls in order to avoid data analysis bias, the analysis of the association of MICA and MICB alleles with MPN was performed by fitting additive generalized linear models with age and gender as covariates [7,17]. Analyses were initially performed independently for each locus using two-field resolution. The analysis was executed through the HIBAG package (version 1.44.0) for Bioconductor (release 3.21) for R for Windows (version 4.5.0) [18]. Associations between MPN and bi-locus haplotypes were analyzed using the haplo.stats package (version 1.9.7) (https://cran.r-project.org/web/packages/haplo.stats/index.html) (accessed on 01 March 2026) through implementation of imputed haplotype score calculation, as described previously [7]. *p*-values below 0.05 were considered significant.

### 2.5. KLRK1 Genotyping and Association Analysis

Five functional single-nucleotide polymorphisms (SNPs) in *KLRK1* (rs2255336, rs1049174, rs2617170, rs2246809, and rs2617160) were genotyped in a subgroup of 35 CALR-mutated MPN patients and 105 healthy controls using a real-time PCR-based allelic discrimination assay. Genotypes were called using standard instrument software. Haplotype associations were evaluated using SNPStats (Institut Català d’Oncologia, Barcelona, Spain) [19], with adjustment for age and sex. Because rs2255336 deviated from the Hardy–Weinberg equilibrium in controls, haplotype analyses were restricted to rs1049174, rs2617160, rs2246809, and rs2617170.

### 2.6. In Silico Modelling

Molecular dynamics simulations (MDSs) of MICA*001:01:01, MICA*004:01:01, and MICA*112:02 proteins in complex with the NKG2D dimer were performed using the AMBER03 force field in GROMACS 2023 [20]. The initial crystal structure was obtained from the Protein Data Bank (PDB ID: 1HYR) [21]. The systems were constructed by modifying the MICA chain (chain C in 1HYR) to match the MICA*004:01:01 and MICA*112:02 sequences retrieved from the IPD-IMGT/HLA Database (release 3.61) [22]. Amino acid substitutions were introduced in PyMOL 2.5.1 [23].

Each complex was solvated with TIP3P water and neutralized by addition of Na^+^ and Cl^−^ counterions. Protonation states at pH 7.0 were estimated using PROPKA [24], and 150 mM NaCl was added to mimic physiological ionic strength. After energy minimization, systems were heated to 310 K in the constant-temperature, constant-volume (NVT) ensemble for 400 ps, followed by production simulations in the isothermal–isobaric (NPT) ensemble at 310 K and 1 atm.

For all simulations, the production phase lasted for 700 ns, and structural snapshots were saved every 20 ps. The root-mean-square deviations (RMSDs) of atomic coordinates for all atoms of the complex were calculated relative to the initial production structure using built-in GROMACS tools. To characterize the effect of the structural changes in MICA variations on the stability of the NKG2D–MICA complex, a detailed analysis of the ligand–receptor interfaces was performed using LigPlot+ v2.3.1 [25,26].

### 2.7. Gene Expression Analyses

We reanalyzed a publicly available single-cell RNA sequencing (scRNA-seq) dataset with genotype-resolved transcriptomes from bone marrow cells of five CALR-mutated essential thrombocythemia (ET) and five CALR-mutated myelofibrosis (MF) patients (GSE117826) [27]. Processed, filtered, and integrated data were handled as a Seurat object, as described previously [6]. Dimensionality reduction was performed using t-SNE [28] in Seurat version 5.4.0 [29]. Cell-type annotation was performed using the celldex (version 1.22.0) and SingleR (version 2.14.0) packages with a hematopoietic reference dataset [30], as described previously [6]. MICA and MICB expression in CALR-mutant versus wild-type HSCs, common myeloid progenitors (CMPs), and megakaryocyte-erythroid progenitors (MEPs) was visualized using dot plots implemented through the SCpubr (version 3.0.1) package [31].

We additionally analyzed an RNA-sequencing dataset of human iPSC-derived megakaryocytes (MKs) edited by CRISPR/Cas9 to harbor heterozygous or homozygous CALR frameshift mutations, alongside an isogenic CRISPR control line (GSE182479) [32]. Processed gene counts were downloaded from GEO and normalized to log2-transformed transcripts per million (TPM). MICA and MICB expression was compared between genotypes using two-sided *t*-tests and visualized with the ggpubr package (v. 0.6.1). Gene set enrichment analysis (GSEA; v4.4.0) was performed on normalized expression data using the standard workflow [33,34] and custom gene sets for antigen processing and presentation pathways, as described previously [4,5,6].

## 3. Results

### 3.1. MICA*004:001 Allele Is Associated with CALR-Mutated MPNs

**Figure 1:**
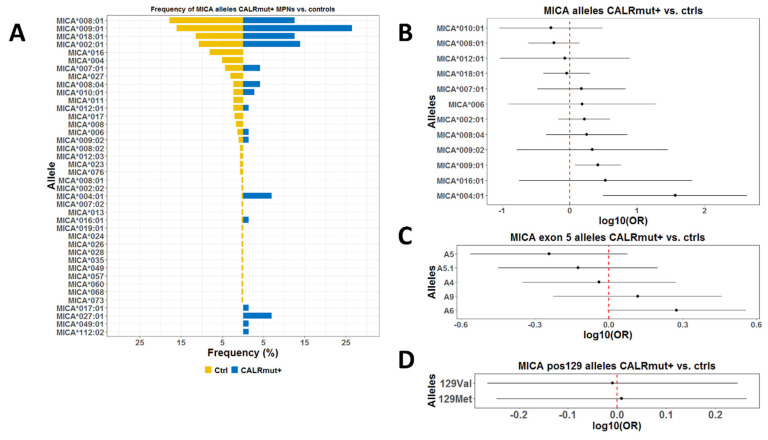
MICA allele distributions and associations in CALR-mutated MPN versus healthy controls. (**A**) Frequencies of identified MICA alleles in CALR-mutated MPN patients and healthy controls. (**B**) Odds ratios (ORs) for association of MICA alleles with CALR-mutated status (age- and sex-adjusted additive generalized linear model). (**C**) ORs for association of MICA exon 5-defined groups with CALR-mutated status (age- and sex-adjusted generalized linear model). (**D**) ORs for association of MICA codon 129-defined groups with CALR-mutated status (age- and sex-adjusted generalized linear model).

**Figure 2:**
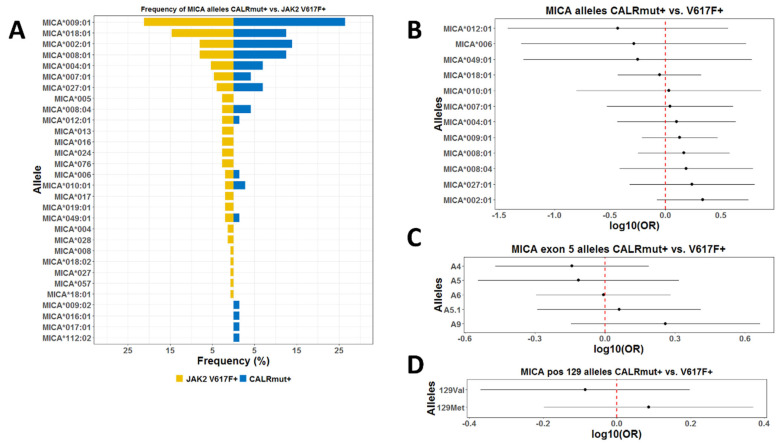
MICA allele distributions and associations in CALR-mutated versus JAK2 V617F-positive MPN. (**A**) Frequencies of identified MICA alleles in CALR-mutated MPN patients and JAK2 V617F-positive MPN patients. (**B**) Odds ratios (ORs) for association of MICA alleles with CALR-mutated status (age- and sex-adjusted additive generalized linear model). (**C**) ORs for association of MICA exon 5-defined groups with CALR-mutated status (age- and sex-adjusted generalized linear model). (**D**) ORs for association of MICA codon 129-defined groups with CALR-mutated status (age- and sex-adjusted generalized linear model).

When we compared the frequencies of MICA alleles in CALR-mutated MPNs vs. healthy controls after adjusting for age and sex, we did not identify any protective MICA allele (Supplementary Tables S1–S3). However, the MICA*004:001 allele was significantly associated with CALR-mutant status (*p* = 0.004; adjusted *p* = 0.047) (Figure 1A,B and Supplementary Table S1). When we tested for any associations with alleles defined by exon 5 or codon 129 polymorphisms, we did not identify any significant associations (Figure 1C,D and Supplementary Tables S2 and S3). When we performed analogous analyses comparing CALR-mutated MPNs vs. JAK2 V617F-positive MPNs, we did not identify any significant associations (Figure 2) (Supplementary Tables S4–S6). Interestingly, we identified a novel MICA allele in a CALR-mutant patient which was annotated as MICA*112:02 (Supplementary Table S1) [35].

### 3.2. MICB*008:001 Allele Shows Nominal Association with CALR-Mutated MPNs

**Figure 3:**
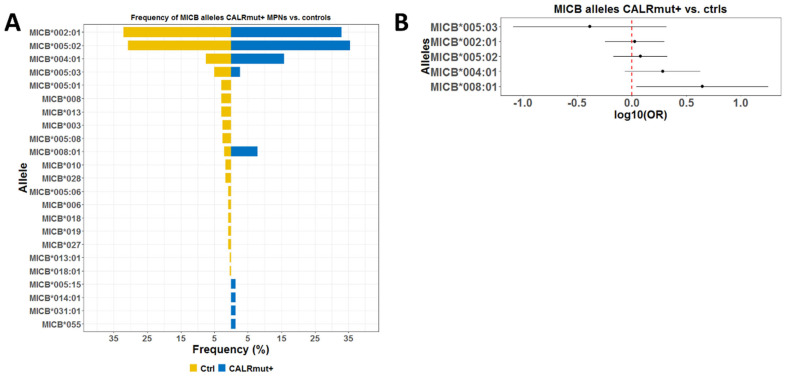
MICB allele distributions and associations in CALR-mutated MPN versus healthy controls. (**A**) Frequencies of identified MICB alleles in CALR-mutated MPN patients and healthy controls. (**B**) Odds ratios (ORs) for association of MICB alleles with CALR-mutated status (age- and sex-adjusted additive generalized linear model).

**Figure 4:**
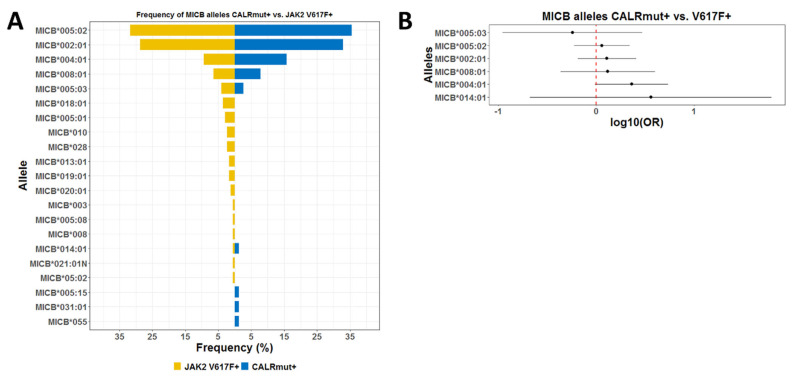
MICB allele distributions and associations in CALR-mutated versus JAK2 V617F-positive MPN. (**A**) Frequencies of identified MICB alleles in CALR-mutated MPN patients and JAK2 V617F-positive MPN patients. (**B**) Odds ratios (ORs) for association of MICB alleles with CALR-mutated status (age- and sex-adjusted additive generalized linear model).

When we searched for association of MICB alleles with CALR-mutated MPNs vs. healthy controls (Figure 3A and Supplementary Table S7), one allele showed a nominal positive association, MICB*008:001 (*p* = 0.037), which was lost after Bonferroni adjustment (*p* = 0.184) (Figure 3B). When we compared CALR-mutated MPNs vs. JAK2 V617F-positive MPNs, no significant associations were found (Figure 4A,B and Supplementary Table S8). Of note, however, we identified two novel MICB alleles in CALR-mutant patients, annotated as MICB*055 and MICB*005:15 (Figure 3A and Figure 4A; Supplementary Table S7) [36].

### 3.3. Haplotype Associations

**Figure 5:**
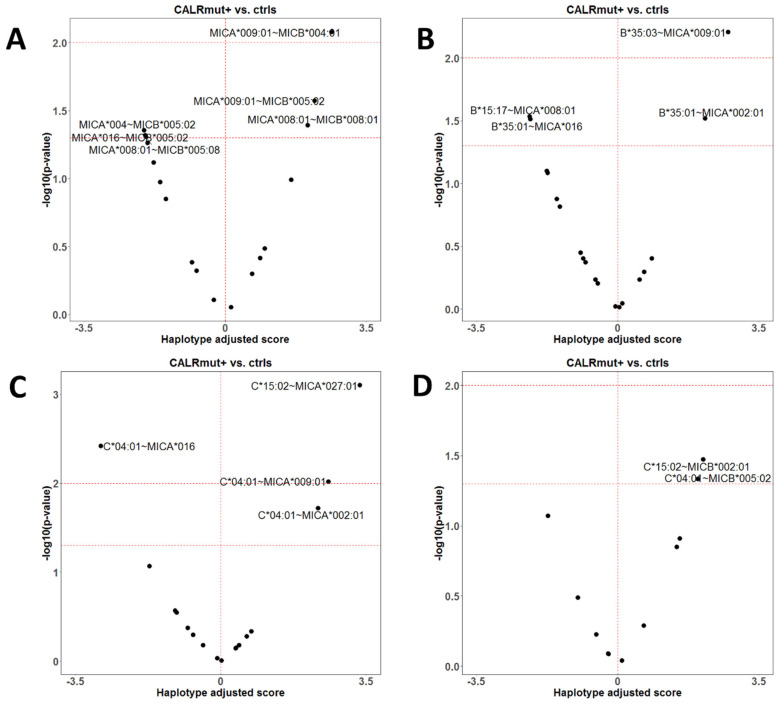
Volcano plots of haplotype associations with CALR-mutated MPN versus healthy controls. (**A**) MICA~MICB haplotypes. (**B**) HLA-B~MICA haplotypes. (**C**) HLA-C~MICA haplotypes. (**D**) HLA-C~MICB haplotypes.

We further tested for biallelic haplotype associations with CALR-mutant status in comparison to healthy controls (Supplementary Tables S9–S15) and JAK2 V617F-mutated MPNs (Supplementary Tables S16–S22). We defined MICA~MICB and single-locus HLA-A/B/C~MICA/MICB haplotypes. Because several alleles and haplotypes were evaluated in a relatively small CALR-mutated cohort, these analyses should be interpreted as exploratory and require independent replication. Analyses that showed nominal associations are shown in Figure 5. The MICA*009:01-MICB*004:001 haplotype was nominally associated with CALR-mutated MPN versus healthy controls (*p* = 0.008) (Figure 5A and Supplementary Table S15). The B*35:01-MICA*002:001 haplotype showed a positive nominal association, whereas the B*35:01-MICA*016 haplotype showed an apparent protective nominal association (Figure 5B). The lowest *p*-value was observed for the HLA-C*15:02-MICA*027:01 haplotype (Figure 5C). Finally, several weaker nominal associations were observed for MICB-containing biallelic haplotypes (Figure 5D).

### 3.4. NKG2D Polymorphisms

In a genotyped subgroup (35 CALR-mutated MPN patients and 105 controls), we assessed the associations of four *KLRK1* SNPs (rs1049174, rs2617160, rs2246809, and rs2617170) with CALR-mutated status. The estimated haplotype frequencies are shown in Table 2 (upper panel). In age- and sex-adjusted haplotype association models, a single haplotype (G-A-G-T; rs1049174-rs2617160-rs2246809-rs2617170) was associated with increased risk of CALR-mutated MPN (Table 2, lower panel).

### 3.5. Molecular Dynamics Simulations

**Figure 6:**
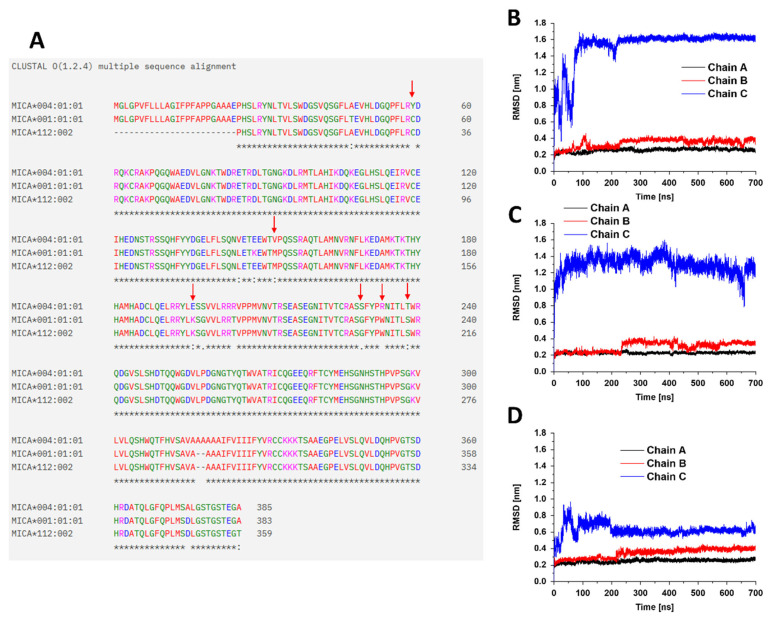
Molecular dynamics simulations of selected MICA variant molecules in complex with NKG2D. (**A**) Clustal-based alignment of protein sequences of selected MICA alleles (MICA*001:01:01, MICA*004:01:01, and MICA*112:02) from the IPD-IMGT/HLA database. The red arrows mark the key amino acid residues defining the two main subgroups of MICA molecules, as discussed in the main text. (**B**) RMSD values from molecular dynamics simulation of the MICA*001:01:01-NKG2D complex. (**C**) RMSD values from molecular dynamics simulation of the MICA*004:01:01-NKG2D complex. (**D**) RMSD values from molecular dynamics simulation of the MICA*112:02-NKG2D complex.

To obtain structural context for selected MICA variants, we performed molecular dynamics simulations of MICA*004:01:01 and MICA*112:02 in complex with NKG2D, using the published MICA*001:001-NKG2D structure as the initial template [21]. The initial structures were modified according to the conventional amino acid alignment (Figure 6A) and then subjected to 700 ns production MD simulations (Figure 6B–D). These simulations were designed to compare conformational behavior and interface contacts in silico; they were not intended to quantify binding affinity or to infer NKG2D-mediated immune recognition in patients. The final 100 ns of each trajectory were subjected to statistical analysis. Five thousand (5000) frames were used for similarity-based clustering of structures via two cluster analysis methods: Jarvis-Patrick [37] and gromos [38], using a cutoff of 0.16 nm. In the former method, clustering was performed based on the coordinates of the entire complex system, while in the second, it was based on the coordinates of the backbone atoms. Clusters with a population of over 1 500 structures were considered representative. Regardless of the method, the MICA*001:01:01-NKG2D complex clustered into only one average (central) structure, whereas MICA*004:01:01-NKG2D clustered into nine structures (with two representative structures using the Jarvis-Patrick method) or 43 structures (with two representative structures using the gromos method), and the MICA*112:02-NKG2D complex clustered into one structure (with one representative structure using the Jarvis-Patrick method) or 22 structures (with one representative structure using the gromos method).

**Figure 7:**
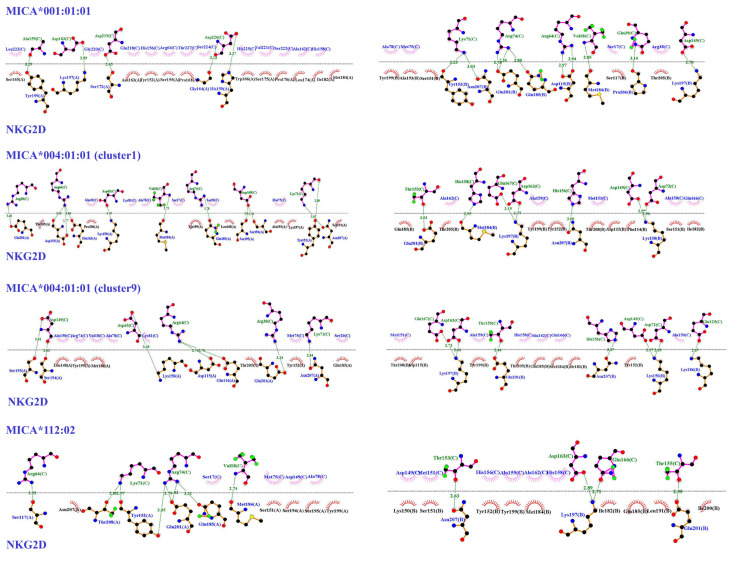
Representative MICA-NKG2D interface contacts observed during last 100 ns of productive molecular dynamics simulations. Hydrogen bonds and salt bridges were identified across the trajectories for MICA*001:01:01, MICA*004:01:01, and MICA*112:02 complexes.

Figure 7 shows interaction maps of the representative clusters of each studied system. The amino acid substitutions defining MICA*004:01:01 and MICA*112:02 do not directly involve residues described as core contact residues in the crystal structure of the MICA*001:01:01-NKG2D complex. Nevertheless, molecular dynamics simulations under physiological conditions indicated allele-specific changes in the interface architecture and identified alternative contacts involving additional amino acids.

### 3.6. Single-Cell RNA-Sequencing Analysis

**Figure 8:**
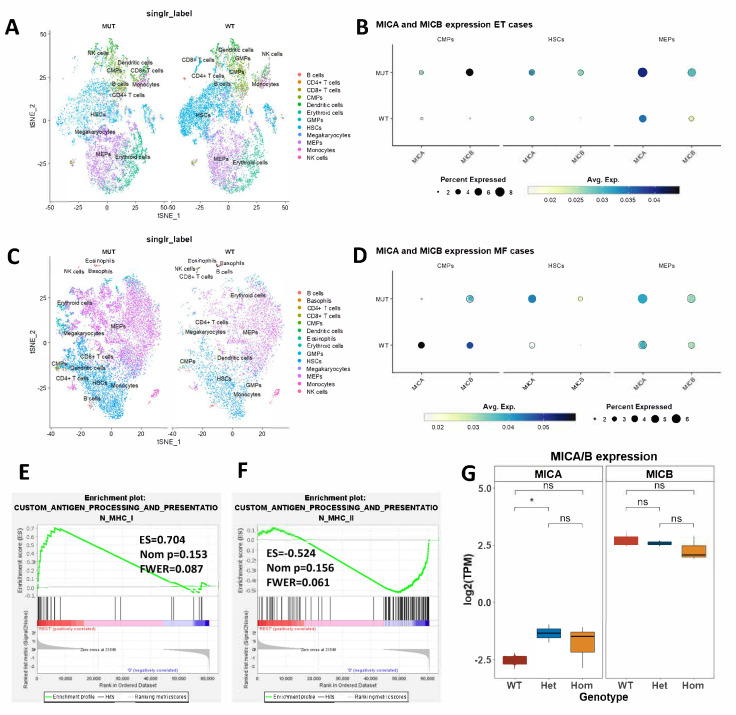
Expression of MICA and MICB in CALR-mutant hematopoietic compartments. (**A**) Two-dimensional scatter plot after t-SNE transformation of integrated scRNA-seq data from five CALR-mutated ET cases. (**B**) Dot plot summarizing MICA and MICB expression in HSCs, CMPs, and MEPs from ET cases stratified by CALR genotype. (**C**) Two-dimensional scatter plot after t-SNE transformation of integrated scRNA-seq data from five CALR-mutated MF cases. (**D**) Dot plot summarizing MICA and MICB expression in HSCs, CMPs, and MEPs from MF cases stratified by CALR genotype. (**E**) GSEA plots of enrichment of MHC-I antigen processing and presentation pathway genes in CALR-mutant versus CALR-wild type MKs. (**F**) GSEA plots of enrichment of MHC-I antigen processing and presentation pathway genes in CALR-mutant versus CALR-wild type MKs. (**G**) MICA and MICB expression in iPSC-derived megakaryocytes (MKs) (control, heterozygous CALR-mutant, and homozygous CALR-mutant). *p*-values are from two-sided *t*-tests. Statistical notation: ns, *p ≥* 0.05; *, *p* < 0.05. Abbreviations: HSCs, hematopoietic stem cells; CMPs, common myeloid progenitors; MEPs, megakaryocyte–erythroid progenitors.

We analyzed publicly available scRNA-seq data with genotype-resolved transcriptomes from five CALR-mutated ET and five CALR-mutated MF patients (GSE117826) [27], enabling direct comparison of MICA and MICB expression between CALR-mutant and wild-type cells within the same microenvironmental context [6]. In ET cases (Figure 8A,B), CALR-mutant HSCs, CMPs, and MEPs showed a higher fraction of cells with detectable MICA and MICB transcripts than the wild type. Similar patterns were observed in MF cases, with higher MICA expression in CALR-mutant HSC and MEP compartments (Figure 8C,D).

We further examined whether CALR gene dosage affects MICA/MICB expression in megakaryocytes using RNA-seq data from CRISPR/Cas9-edited human iPSC-derived megakaryocytes (MKs) (Figure 8E–G) [32]. GSEA showed relative enrichment of MHC-I and MHC-II antigen processing and presentation pathways in CALR-mutant MKs (Figure 8E,F). Heterozygous CALR-mutant MKs showed higher MICA expression relative to controls, which is compatible with a cell-intrinsic effect of mutant CALR on MICA regulation, whereas MICB expression was not materially altered (Figure 8G).

## 4. Discussion

Immunoediting is increasingly recognized in early MPN pathogenesis [1,39]. Adaptive immune recognition of MPN stem cells can be driven by both driver and passenger mutations and is expected to be HLA-restricted. Consistent with this, several studies have identified HLA class I alleles that are under-represented in JAK2 V617F- or CALR-mutated MPN, suggesting partial protection [3,4,6]. Because such effects are not absolute, additional immunogenetic factors may contribute to immune control. We previously observed associations implicating the NKG2D ligand axis in JAK2 V617F-positive MPN (MICA*008:01 and MICA*016 as potentially protective alleles and MICB*018:01) [7]. In the current study, we evaluated whether the NKG2D-MICA/MICB axis also contributes to susceptibility to CALR-mutated MPN.

We genotyped MICA and MICB in 43 Bulgarian patients with CALR-mutated MPN and compared allele frequencies with those in 85 JAK2 V617F-positive MPN patients and 156 healthy Bulgarian controls [7]. We identified MICA*004:001 as an allele associated with increased CALR-mutated MPN risk versus controls. MICA*004 is among the most common alleles in European populations [40,41,42,43], and despite our modest sample size, we also observed three previously unreported MICA/MICB alleles [35,36]. Because MICA polymorphisms can influence NKG2D binding, we performed molecular dynamics simulations to explore potential structural correlates. Prior work has classified MICA variants into higher- versus lower-affinity types based on linked polymorphic residues [44,45]. In this framework, MICA*004:001 is categorized as a higher-affinity (type I) variant, whereas MICA*001:01 and MICA*112:02 fall within the lower-affinity (type II) group. Consistent with this classification, 700 ns simulations showed similar dynamics for MICA*001:01 and MICA*112:02 that differed from MICA*004:001. These structural observations are hypothesis-generating only. They do not demonstrate altered NKG2D binding affinity, NK-cell or CD8+ T-cell activation, cytokine production, or immune recognition in patients.

The MICA*001:01:01-NKG2D complex demonstrated conformational homogeneity, with 100% of the trajectory classified into a single cluster with a cutoff of 0.16 nm. Cluster analysis revealed greater conformational dynamics in the MICA*004:01:01-NKG2D complex, which formed nine distinct clusters at the same cutoff. The presence of two dominant populations (Cluster 1 and Cluster 9), which were nearly equal in size (~30% and ~29% of the frames, respectively), suggests that the simulated MICA*004:01:01-NKG2D complex sampled two main conformational states. In contrast, the two amino acid differences in the MICA*112:02-NKG2D model were associated with a more stable trajectory that was dominated by one representative conformation. These computational differences should be interpreted as structural observations rather than evidence of altered biological function.

A detailed analysis of the MICA*001:01:01-NKG2D complex shows a specific network of hydrogen bonds, primarily involving acidic residues such as Asp (aspartic acid) on the MICA (Chain C) and basic or polar residues (Lys and Ser) on the NKG2D (Chains A and B). The binding of Chain A to Chain C is primarily stabilized by hydrogen bonds and salt bridges. Notable interactions include the polar coordination between His159(A) and Asp226(C), and the electrostatic coupling of Lys197(A) with Asp163(C); Ser172(A) forms a hydrogen bond with Asp219(C). Interaction analysis of the Chain B sub-unit reveals a distinct binding mode. Hydrogen bonds are observed between Tyr152(B) and Lys71(C), Arg64(C) and Arg74(C). Furthermore, a salt-bridge network is established by Asp115(B) with Arg64(C) and Arg74(C). After 700 ns of simulation, interactions of the two chains of NKG2D with MICA remain asymmetric.

The mutations in the MICA*112:02 allele do not change the binding pattern in the MICA*112:02-NKG2D complex. The loss of the Thr24 hydroxyl group and the reversal of the Lys125 charge cause a conformational adaptation. The interaction between Arg74 and Lys71 of MICA*112:02 forms persistent contacts with Tyr152, Gln185 and Glu201 of NKG2D. Tyr152(A) and Arg74(C) remain the factors in high structural convergence and populate a single conformational cluster.

The substitution of eleven amino acids in the MICA*004:01:01 allele induced significant structural heterogeneity, resulting in two dominant clusters (Cluster 1 and Cluster 9). The representative conformations of both clusters show a significant shift in the bonding network compared with the MICA*001:01:01-NKG2D complex. The loss of native contacts caused by a high mutational rate is compensated by the formation of alternative salt bridges and polar interactions. Key residues, including Arg64 and Asp149 of MICA*004:01:01, interact with Asp115 and Glu116 of NKG2D. The NKG2D interface in this alternative state is stabilized by Thr155 and His156 of MICA through coordinated interactions with Lys197 and Glu201 of NKG2D.

The comparative data indicate that Tyr152 of the NKG2D dimer and the basic residues Arg74 and Lys71 of MICA constitute important recognition elements required for assembly of the complex. The two-residue difference in MICA*112:02 appears structurally well tolerated and preserves key hydrogen-bonding interactions involving Tyr152 (on both Chains A and B) and Arg74 of Chain C. In contrast, the LigPlot analysis suggests that MICA*004:01:01 is associated with a broader reconfiguration of protein–receptor interactions. The transition from a lysine-rich cationic interface in MICA*001:01:01 to a more glutamate-rich anionic interface in MICA*004:01:01, together with loss of specific hydrogen bonds, is compatible with distinct simulated interface geometry. However, this modeling result does not demonstrate altered NKG2D binding affinity, cytotoxicity, cytokine production, or pro-inflammatory signaling.

NKG2D activity is influenced not only by MICA/MICB polymorphism but also by functional variation in KLRK1, which affects NKG2D expression and lymphocyte cytotoxicity [46,47]. In our cohort, one KLRK1 haplotype (G-A-G-T; rs1049174-rs2617160-rs2246809-rs2617170) showed an exploratory association with increased risk of CALR-mutated MPN and has previously been reported to confer higher cytotoxic activity in CD8+ T and NK cells [46,48]. This haplotype has also been linked to susceptibility to rheumatoid arthritis [49], consistent with the broader role of NKG2D in chronic inflammatory and autoimmune disorders [50] and with the inflammatory state in MPNs that contributes to disease progression [51,52]. Notably, functional studies on MPN have demonstrated quantitative and qualitative NK-cell defects and modulation by interferon-alpha or ruxolitinib therapy [53,54]. Together, these observations suggest that inherited NKG2D-related variation may interact with disease-associated NK-cell dysfunction, but direct functional assays are required to determine whether this translates into altered immune surveillance or inflammation in CALR-mutated MPN.

MICA/MICB expression represents an additional regulatory layer for the NKG2D axis. By reanalyzing scRNA-seq data from CALR-mutated ET and MF, we found that only a minority of HSCs, CMPs, and MEPs had detectable MICA or MICB transcripts, but the fraction and expression level were higher in CALR-mutant compared with wild-type cells. Because immune selection may extend beyond the stem/progenitor compartment, we analyzed an RNA-seq dataset of iPSC-derived megakaryocytes with engineered CALR mutations. Heterozygous CALR-mutant megakaryocytes exhibited higher MICA expression than controls, whereas MICB was not appreciably changed. These findings support the possibility that a subset of CALR-mutant megakaryocytes—and potentially platelets—may engage NKG2D-expressing lymphocytes independently of effective MHC-I presentation. This interpretation is consistent with observations of increased platelet-CD8+ T-cell aggregates in MPN and immunoregulatory platelet phenotypes enriched in CALR-mutant disease [55,56], but it remains to be validated at the protein and functional levels.

Overall, our data support a modest and exploratory link between the NKG2D-MICA/MICB axis and the immunogenetic landscape of CALR-mutated MPN. The association of MICA*004:001 and a KLRK1 haplotype, together with increased MICA/MICB transcription in CALR-mutant hematopoietic compartments, is compatible with a role for immune selection, but these findings do not establish a direct mechanistic contribution to disease pathogenesis. From a translational perspective, therapies that increase surface expression of NKG2D ligands or counteract ligand shedding could theoretically enhance innate immune recognition [57,58,59]. However, such approaches would require careful preclinical evaluation because excessive or chronic activation of NK-cell and CD8+ T-cell pathways may also contribute to inflammatory pathology. Thus, therapeutic modulation of this axis should be regarded as a testable hypothesis rather than a conclusion of the current study.

Several limitations should be considered when interpreting the present results. First, the CALR-mutated cohort was small (n = 43), and the KLRK1 analysis was performed in an even smaller subgroup (n = 35). Given the number of alleles, SNPs, and haplotypes tested, some nominal associations may represent false-positive findings. Although the MICA*004:001 allele remained significant after Bonferroni correction in the comparison with healthy controls, the haplotype-level findings should be considered exploratory and require validation in larger, independent cohorts with harmonized ancestry and clinical annotation. Second, protein-level and functional validation of the NKG2D-MICA/MICB axis was not available in the analyzed cohort. Specifically, soluble MICA and MICB concentrations were not measured, and viable patient-derived material was not available for systematic assessment of surface MICA/MICB expression, NK-cell or CD8+ T-cell cytotoxicity, degranulation, or cytokine production, including IL-6, IL-10, IL-2, and IFN-gamma. Therefore, although the genetic, structural, and transcriptomic data are compatible with involvement of this axis in CALR-mutated MPN, they do not directly prove a pro-inflammatory functional interaction between MICA/MICB and NKG2D-expressing lymphocytes. Future studies should integrate MICA/MICB and KLRK1 genotyping with plasma soluble-ligand quantification, flow-cytometric analysis of ligand expression in CD34+ and megakaryocytic compartments, and functional assays of NK-cell and CD8+ T-cell activation.

## Figures and Tables

**Table 1:** Demographic and clinical characteristics of study participants.

Parameter	Group			*p*-Value
	CALR-Mutated MPNs	JAK2 V617F-Positive MPNs	Healthy Controls	
Sex				0.2233 *
Male	23	38	61	
Female	20	47	95	
Total	43	85	156	
Age				<2.2 × 10^−16^ †
Median	66	71	32	
Range	30–87	32–96	20–99	
Diagnosis				
ET	16	30	NA	<2.2 × 10^−16^ ‡
MF post-ET	2	1	NA	
PMF	14	11	NA	
PV	0	37	NA	
MF post-PV	0	2	NA	
MPN; NOS	11	4	NA	
Total	43	85	NA	

Abbreviations: ET, essential thrombocythemia; PMF, primary myelofibrosis; PV, polycythemia vera; MPN, NOS, myeloproliferative neoplasm, not otherwise specified; NA, not applicable. *p*-values: * chi-squared test across three groups; † Kruskal–Wallis test across three groups; ‡ chi-squared test across two groups.

**Table 2:** KLRK1 (NKG2D) haplotype frequencies and association with CALR-mutated MPN using SNPStats web-server analysis.

Haplotype Frequency Estimation (*n* = 140)								
	rs1049174 (NKC-3)	rs2617160 (NKC-7)	rs2246809 (NKC-9)	rs2617170 (NKC-11)	Total	Healthy Controls	CALR-Mutated MPNs	Cumulative Frequency
1	C	T	G	C	0.563	0.5899	0.4848	0.563
2	G	A	A	T	0.185	0.2006	0.1278	0.7482
3	G	A	G	T	0.172	0.1564	0.2273	0.9197
4	C	T	G	T	0.044	0.0289	0.0847	0.9635
5	G	T	G	T	0.012	0.0075	0.0303	0.9757
6	C	A	G	C	0.008	0.0049	0	0.9836
7	G	T	G	C	0.007	0.0049	0.0145	0.991
8	G	T	A	T	0.006	0.0068	0	0.9969
9	C	A	G	T	0.003	NA	0.0155	1
10	C	A	A	C	0	NA	0.015	1
Haplotype association with response (*n* = 140, adjusted by sex and age)								
	rs1049174 (NKC-3)	rs2617160 (NKC-7)	rs2246809 (NKC-9)	rs2617170 (NKC-11)	Frequency	OR (95% CI)	*p*-Value	
1	C	T	G	C	0.563	1	---	
2	G	A	A	T	0.186	0.85 (0.29–2.53)	0.78	
3	G	A	G	T	0.171	3.61 (1.16–11.26)	0.029	
4	C	T	G	T	0.044	3.75 (0.60–23.42)	0.16	
5	G	T	G	T	0.013	3.65 (0.15–91.62)	0.43	
Rare	*	*	*	*	0.024	9.08 (0.96–86.05)	0.057	

The upper panel represents comparison of the frequencies in healthy controls and CALR-mutated MPN patients using chi-squared testing without adjustment. The lower panel represents comparison of the frequencies in healthy controls and CALR-mutated MPN patients using logistic regression adjusted for sex and age. *p*-values below 0.05 were considered statistically significant. Abbreviations: OR, odds ratio; NA, not applicable. Abbreviations: * any nucleotide.

## Data Availability

Genotype data for Bulgarian subjects are available from the authors upon reasonable request and after signing a material transfer agreement. All other datasets are publicly available and can be accessed as described in the main text.

## Supplementary Materials

The following supporting information can be downloaded at: https://www.mdpi.com/article/10.3390/cancers18132052/s1, Supplementaty Table S1. Association of MICA alleles with CALRmut+ MPNs vs. healthy controls. Additive generalized linear model with age (continuous) and gender (binary) as covariables. Supplementaty Table S2. Association of MICA exon 5 alleles with CALRmut+ MPNs vs. healthy controls. Additive generalized linear model with age (continuous) and gender (binary) as covariables. Supplementaty Table S3. Association of MICA codon 129 alleles with CALRmut+ MPNs vs. healthy controls. Additive generalized linear model with age (continuous) and gender (binary) as covariables. Supplementaty Table S4. Association of MICA alleles with CALRmut+ MPNs vs. JAK2 V617F+ MPNs. Additive generalized linear model with age (continuous) and gender (binary) as covariables. Supplementaty Table S5. Association of MICA exon 5 alleles with CALRmut+ MPNs vs. JAK2 V617F+ MPNs. Additive generalized linear model with age (continuous) and gender (binary) as covariables. Supplementaty Table S6. Association of MICA codon 129 alleles with CALRmut+ MPNs vs. JAK2 V617F+ MPNs. Additive generalized linear model with age (continuous) and gender (binary) as covariables. Supplementaty Table S7. Association of MICB alleles with CALRmut+ MPNs vs. healthy controls. Additive generalized linear model with age (continuous) and gender (binary) as covariables. Supplementaty Table S8. Association of MICB alleles with CALRmut+ MPNs vs. JAK2 V617F+ MPNs. Additive generalized linear model with age (continuous) and gender (binary) as covariables. Supplementaty Table S9. Association of HLA-A~MICA haplotypes with CALRmut+ MPNs vs. healthy controls. Additive generalized linear model with age (continuous) and gender (binary) as covariables. Supplementaty Table S10. Association of HLA-A~MICB haplotypes with CALRmut+ MPNs vs. healthy controls. Additive generalized linear model with age (continuous) and gender (binary) as covariables. Supplementaty Table S11. Association of HLA-B~MICA haplotypes with CALRmut+ MPNs vs. healthy controls. Additive generalized linear model with age (continuous) and gender (binary) as covariables. Supplementaty Table S12. Association of HLA-B~MICB haplotypes with CALRmut+ MPNs vs. healthy controls. Additive generalized linear model with age (continuous) and gender (binary) as covariables. Supplementaty Table S13. Association of HLA-C~MICA haplotypes with CALRmut+ MPNs vs. healthy controls. Additive generalized linear model with age (continuous) and gender (binary) as covariables. Supplementaty Table S14. Association of HLA-C~MICB haplotypes with CALRmut+ MPNs vs. healthy controls. Additive generalized linear model with age (continuous) and gender (binary) as covariables. Supplementaty Table S15. Association of MICA~MICB haplotypes with CALRmut+ MPNs vs. healthy controls. Additive generalized linear model with age (continuous) and gender (binary) as covariables. Supplementaty Table S16. Association of HLA-A~MICA haplotypes with CALRmut+ MPNs vs. JAK2 V617F+ MPNs. Additive generalized linear model with age (continuous) and gender (binary) as covariables. Supplementaty Table S17. Association of HLA-A~MICB haplotypes with CALRmut+ MPNs vs. JAK2 V617F+ MPNs. Additive generalized linear model with age (continuous) and gender (binary) as covariables. Supplementaty Table S18. Association of HLA-B~MICA haplotypes with CALRmut+ MPNs vs. JAK2 V617F+ MPNs. Additive generalized linear model with age (continuous) and gender (binary) as covariables. Supplementaty Table S19. Association of HLA-B~MICB haplotypes with CALRmut+ MPNs vs. JAK2 V617F+ MPNs. Additive generalized linear model with age (continuous) and gender (binary) as covariables. Supplementaty Table S20. Association of HLA-C~MICA haplotypes with CALRmut+ MPNs vs. JAK2 V617F+ MPNs. Additive generalized linear model with age (continuous) and gender (binary) as covariables. Supplementaty Table S21. Association of HLA-C~MICB haplotypes with CALRmut+ MPNs vs. JAK2 V617F+ MPNs. Additive generalized linear model with age (continuous) and gender (binary) as covariables. Supplementaty Table S22. Association of MICA~MICB haplotypes with CALRmut+ MPNs vs. JAK2 V617F+ MPNs. Additive generalized linear model with age (continuous) and gender (binary) as covariable.

## Abbreviations

The following abbreviations are used in this manuscript:

ET	Essential thrombocythemia
PMF	Primary myelofibrosis
PV	Polycythemia vera
MPN	Myeloproliferative neoplasm
NK	Natural killer
HLA	Human leukocyte antigen
SNP	Single-nucleotide polymorphism
iPSC	Induced pluripotent stem cell
MK	Megakaryocyte
scRNA-seq	Single-cell RNA sequencing
MDS	Molecular dynamics simulation
